# Standardized *Rhodiola rosea* injection for left ventricular remodeling and inflammation in patients with HFrEF: a systematic review and meta-analysis

**DOI:** 10.3389/fphar.2025.1536686

**Published:** 2025-03-03

**Authors:** Xuqin Du, Xiaorong Wang, Ruodai Zhang, Yong Chen, Qian Chen, Jing Yao, Lipeng Shi, Yi Ren

**Affiliations:** ^1^ School of Traditional Chinese Medicine, Chongqing University of Chinese Medicine, Chongqing, China; ^2^ College of Traditional Chinese Medicine, Chongqing Medical University, Chongqing, China; ^3^ Department of Classic Traditional Chinese Medicine, Chongqing Traditional Chinese Medicine Hospital, Chongqing, China

**Keywords:** standardized *Rhodiola rosea* injection, heart failure with reduced ejection fraction (HFrEF), left ventricular remodeling (LVR), inflammation, systematic review, meta-analysis

## Abstract

**Background:**

Heart failure with reduced ejection fraction (HFrEF) affects a substantial portion of the global population, with left ventricular remodeling (LVR) and inflammation identified as key contributors to disease progression. Standardized *Rhodiola rosea* Injection (SRRI) is a pharmacopoeia-based botanical drug preparation derived from *Rhodiola rosea*, widely used in China for heart failure treatment. It is standardized in composition and quality control, with known antioxidant, anti-inflammatory, and anti-fibrotic properties. However, comprehensive evaluations of SRRI’s effects on LVR and inflammatory mediators in HFrEF patients are limited.

**Purpose:**

To evaluate the effects of SRRI on LVR and inflammatory mediators in patients with HFrEF.

**Methods:**

A systematic review and meta-analysis were conducted following PRISMA and Cochrane guidelines. Eight databases were searched for randomized controlled trials (RCTs) on SRRI in HFrEF treatment with studies identified from inception to 31 October 2024. Quality assessment of the included studies was conducted using the Cochrane Collaboration’s risk of bias tool and the modified Jadad scale. Statistical analysis was performed using Stata version 17.0, with sensitivity analyses conducted by sequentially excluding studies to assess the robustness of findings. Publication bias was evaluated using Egger’s test.

**Results:**

Twenty-five RCTs with 2,325 participants were included. SRRI significantly improved LVR, indicated by increased LVEF (MD = 6.81, 95% CI: 5.71 to 7.91, *P* < 0.00001), reduced LVEDD (MD = −4.37, 95% CI: −5.42 to −3.33, *P* < 0.00001), and decreased LVESD (MD = −4.48, 95% CI: −5.42 to −3.58, *P* < 0.00001). Additionally, SRRI effectively reduced inflammatory mediators, including TNF-α (MD = −10.37, 95% CI: −12.96 to −7.78, *P* < 0.00001), IL-6 (MD = −6.99, 95% CI: −8.88 to −5.11, *P* < 0.00001), and hs-CRP (MD = −2.58, 95% CI: −3.37 to −1.79, *P* < 0.00001). SRRI also significantly reduced BNP (MD = −105.10, 95% CI: −132.29 to −77.90, *P* < 0.00001) and NT-pro BNP (MD = −415.95, 95% CI: −553.00 to −278.89, *P* < 0.00001). Clinical effectiveness was improved, with no significant increase in adverse reactions (RR = 0.86, 95% CI: 0.59 to 1.25, *P* = 0.44). Sensitivity analyses confirmed the robustness of these findings, and no significant publication bias was detected.

**Conclusion:**

SRRI appears to effectively enhance LVR, reduce inflammatory mediators, and improve clinical effectiveness in HFrEF patients while maintaining a favorable safety profile. However, the current evidence is limited by methodological shortcomings, and further well-designed, multicenter RCTs are needed to validate these findings, especially in diverse populations and over long-term treatment durations.

**Systematic Review Registration:**

https://www.crd.york.ac.uk/PROSPERO/display_record.php?RecordID=603884, Identifier CRD42024603884.

## 1 Introduction

Heart failure (HF) affects approximately 26 million people worldwide, significantly diminishing quality of life and socioeconomic status while imposing substantial burdens on global healthcare systems (Savarese et al., 2023). Heart failure with reduced ejection fraction (HFrEF), a major subtype of HF that accounts for roughly 50% of cases, is characterized by a left ventricular ejection fraction of less than 40% and pathological dilation of the left ventricle, referred to as left ventricular remodeling (LVR) (Gronda et al., 2020; Hahn et al., 2024). This remodeling is a critical factor contributing to the worsening of cardiac function (Konstam et al., 2011). While the exact pathophysiological mechanisms underlying HF remain unclear, LVR and chronic inflammation are widely recognized as key drivers in the development and progression of HF, and they are significantly associated with poor clinical outcomes (Smart and Madhur, 2023; Zhang and Dhalla, 2024). Numerous studies have shown that reversing LVR and suppressing inflammation can decelerate the progression of HF and improve clinical prognosis (Alcaide et al., 2024; Lugrin et al., 2023; Yin et al., 2024).

Patients with HFrEF experience structural and functional changes in the left ventricle, which closely correlate with the progression of clinical symptoms and an increased risk of adverse cardiovascular events (Canty, 2022). Considerable efforts have been dedicated to identifying optimal treatments that can improve LVR and reduce inflammation, with the aim of alleviating symptoms and enhancing long-term outcomes (Adhyapak, 2022; Nishida et al., 2024). Angiotensin-converting enzyme inhibitors (ACEIs), angiotensin receptor blockers (ARBs), β-blockers, and aldosterone antagonists are commonly used due to their proven benefits, which include improving LVR, suppressing inflammatory responses, enhancing quality of life, and reducing the incidence of adverse clinical outcomes (Crea, 2023; Kittleson, 2024). For a considerable period, the combined use of these medications has been considered the standard treatment approach for patients with HFrEF (Khan et al., 2023). However, the potential long-term side effects of these drugs have raised safety concerns, prompting a reevaluation of their use (Moloce et al., 2022). Consequently, there is an ongoing need to explore new therapeutic agents that can effectively improve LVR and suppress inflammation to enhance clinical outcomes and quality of life for patients with HFrEF.

Standardized *Rhodiola rosea* Injection (SRRI), a pharmacopoeia-based botanical drug derived from the roots and rhizomes of *Rhodiola rosea*, is extensively used in China for the clinical treatment of cardiovascular diseases, particularly HF (Zong et al., 2023). SRRI is a standardized botanical drug preparation with a well-defined phytochemical profile, primarily standardized for its active metabolite, salidroside. Its pharmacological actions include antioxidant, anti-inflammatory, anti-fibrotic effects, and improvement of cardiac function (Sun et al., 2023). Animal studies have shown that salidroside enhances cardioprotection in acutely exhausted rats by increasing the expression of p-ERK and reducing p-p38 in the mitogen-activated protein kinases (MAPKs) signaling pathway, thereby delaying or mitigating apoptosis induced by oxidative stress (Qi et al., 2017). Research by Chen et al. indicated that salidroside downregulates the expression levels of TNF-α, TGF-β1, IL-1β, and Bax, and upregulates Bcl-2, VEGF, Akt, and eNOS, exerting anti-inflammatory effects and alleviating myocardial fibrosis and remodeling after myocardial infarction (Chen et al., 2019). Furthermore, salidroside enhances the contractile capacity of cardiac myocytes and activates the renin-angiotensin-aldosterone system in rats with HF, contributing to its beneficial effects on cardiac function and ventricular remodeling (Wu et al., 2016). Thus, SRRI is widely utilized in China as an adjunctive therapy for HF. However, comprehensive evaluations of SRRI’s effects on LVR and inflammatory mediators in patients with HFrEF are limited. Given the stringent quality control and standardization processes applied in its production, further research is needed to establish its efficacy and safety profile in broader populations. This systematic review and meta-analysis aim to provide a rigorous evaluation of SRRI in the treatment of HFrEF, focusing on its impact on LVR and inflammatory mediators.

## 2 Methods

This systematic review and meta-analysis were conducted following the guidelines of the Cochrane Collaboration and the Preferred Reporting Items for Systematic Reviews and Meta-Analyses (PRISMA) (Hutton et al., 2015) (Supplementary Material S1). The study protocol was registered on PROSPERO (ID: CRD42024603884).

To ensure the accuracy of the study, these analyses utilized the consensus statement on the phytochemical characteristics of botanical extracts (ConPhyMP) as a reference for reporting SRRI. We also adhered to the guidelines for the scientific nomenclature and standardization of botanical drug ingredients. The botanical drug SRRI used in this study is derived from *Rhodiola rosea* L. [Crassulaceae; Rhodiolae roseae radix et rhizoma], validated taxonomically through the POWO database (http://www.plantsoftheworldonline.org).

### 2.1 Search strategy

We conducted a comprehensive search across eight electronic databases for randomized controlled trials (RCTs): PubMed, Embase, Cochrane Library, Web of Science, Wanfang Database, China Biological Medicine Database (CBM), China Science and Technology Journal Database (VIP), and China National Knowledge Infrastructure (CNKI), covering all entries from database inception through 31 October 2024. No restrictions were applied regarding language or publication date. The search terms included “*Rhodiola rosea*,” “Large *Rhodiola rosea*,” “Dazhu Hongjingtian,” “chronic heart failure,” “heart failure with reduced ejection fraction,” “heart failure,” and “HFrEF.” Additionally, we manually reviewed the reference lists of all identified studies to locate any relevant studies that may have been missed. Detailed search strategies for each database are provided in Supplementary Material S2.

### 2.2 Inclusion and exclusion criteria

The inclusion criteria were as follows: (1) Studies had to be RCTs; other study types were excluded. (2) Participants were required to be aged 18 years or older with a LVEF of less than 40% and classified as NYHA functional class II–IV. (3) The control group received standard heart failure treatment per clinical guidelines, while the intervention group was treated with both SRRI and standard therapy (ST). (4) Primary outcomes included measures of LVR (LVEF, LVEDD, LVESD) and inflammatory mediators (TNF-α, IL-6, hs-CRP).

Exclusion criteria were as follows: (1) Non-randomized studies (e.g., case reports, cohort studies, observational studies, or animal studies). (2) Studies with incomplete or non-extractable data, including those lacking key outcome measures (LVEF, LVEDD, LVESD, TNF-α, IL-6, hs-CRP) or where data could not be reliably extracted for analysis. (3) Duplicate publications or studies with overlapping patient populations. (4) Severe comorbidities in participants, including but not limited to advanced renal failure, severe liver disease, malignancy, or other conditions that could significantly influence the outcomes or safety of the intervention. (5) Studies where the control group did not receive standard treatment for heart failure as per current clinical guidelines.

### 2.3 Data extraction and quality assessment

Two reviewers (XD and LS) independently reviewed and extracted data from each included study. The extracted information included the first author, publication year, article title, sample size, gender distribution, mean age, disease duration, treatment measures for both the intervention and control groups (including dosage, duration, and method of administration). Outcome measures included LVR indicators (LVEF, LVEDD, LVESD), inflammatory mediators (TNF-α, IL-6, hs-CRP), clinical effectiveness, BNP, NT-pro BNP, and adverse reactions. To evaluate the risk of bias in the included studies, we used the Cochrane Collaboration’s tool, which assesses risk in several domains: randomization, allocation concealment, blinding, completeness of outcome data, selective reporting, and other potential sources of bias (Cumpston et al., 2019). Each study’s risk of bias was categorized as low, unclear, or high. We also used the modified Jadad scale to assess study quality, which considers random sequence generation, allocation concealment, blinding, and withdrawals and dropouts, with scores ranging from 1 to 7 (Lunny et al., 2022). Studies scoring between 1 and 3 were considered low quality, while scores between 4 and 7 indicated high quality. Any disagreements were resolved by involving a third reviewer (YR).

### 2.4 Statistical analysis

All data were analyzed using Stata version 17.0. For dichotomous outcomes, we calculated risk ratios (RR) with 95% confidence interval (CI). For continuous outcomes, mean differences (MD) with 95% CI were used. Heterogeneity across studies was assessed using the *I* ^*2*^ statistic; an *I* ^*2*^ of 50% or below indicated low heterogeneity, and in such cases, a fixed-effect model was applied. For higher heterogeneity, a random-effects model was employed. We also performed subgroup analyses based on differences in treatment duration to investigate factors that may influence outcomes.

Sensitivity analyses were conducted by removing individual studies from the analysis to test the robustness of the findings. If the sensitivity analysis revealed no significant changes, the meta-analysis results were considered robust. Conversely, if sensitivity analysis altered the conclusions, the reliability of the results was deemed low, and caution was advised in interpreting these findings. Publication bias was quantitatively assessed using Egger’s test.

## 3 Results

### 3.1 Search results and study characteristics

**FIGURE 1:**
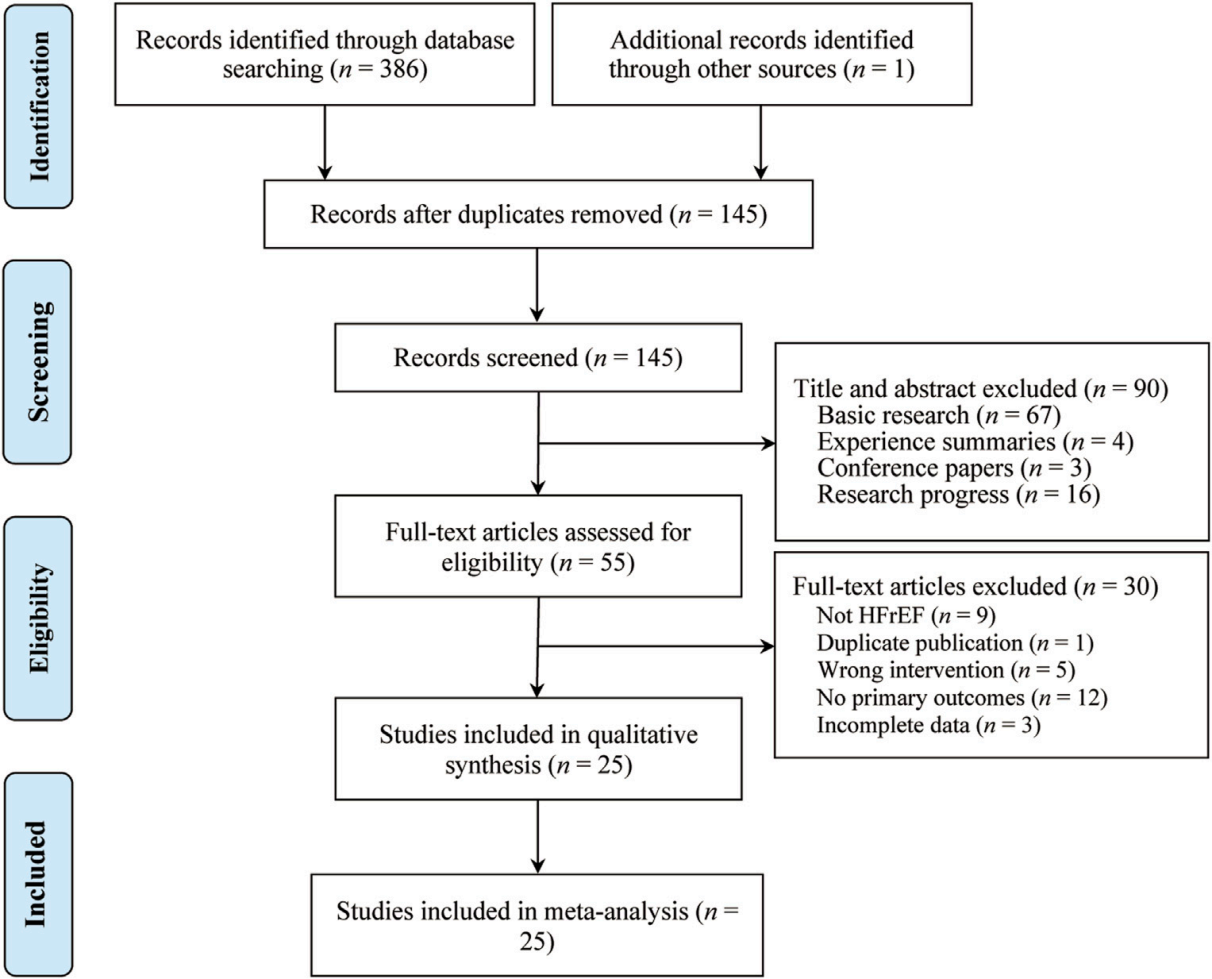
The PRISMA study flowchart.

A total of 387 studies were identified, from which 25 RCTs involving 2,325 participants met the inclusion criteria and were included in the analysis (Figure 1). All 25 RCTs were conducted in China and published between 2011 and 2023 (Chen, 2021; Chen et al., 2016; Dong et al., 2023; Fan et al., 2017; Guan, 2020; Hu et al., 2018; Hua et al., 2017; Jin and Li, 2016; Lai et al., 2019; Li and Liang, 2020; Lu et al., 2020; Ning et al., 2017; Qi et al., 2016; Shen et al., 2017; Song et al., 2020; Tian et al., 2019; Tian et al., 2017; Wang and Liu, 2018; Xie and Wen, 2016; Xu, 2019; Yang, 2019; Zhang and Zhang, 2011; Zhang et al., 2018; Zhang et al., 2020; Zong et al., 2014). Sample sizes ranged from 23 to 110 participants, and treatment durations varied from 10 to 30 days. Control groups received standard heart failure treatments as recommended by clinical guidelines, including digitalis preparations, ACEIs, ARBs, β-blockers, and diuretics. The treatment groups received SRRI in combination with these standard treatments. Baseline characteristics showed no significant differences between the treatment and control groups. The primary outcome measures included LVEF (25 studies), LVEDD (21 studies), LVESD (15 studies), TNF-α (6 studies), IL-6 (7 studies), and hs-CRP (9 studies). Secondary outcomes included clinical effectiveness (21 studies), BNP (9 studies), NT-pro BNP (11 studies), and adverse reactions (12 studies). Table 1 outlines the basic characteristics of the included studies.

**TABLE 1:** Study characteristics.

Included studies	Sample size		Sex (M/F)		Mean age (years)		Course of disease (years)		Interventions		Treatment duration	Outcomes
	T	C	T	C	T	C	T	C	T	C		
Chen. (2021)	40	40	19/21	25/15	58.70±3.19	66.35±8.12	6.11±1.07	5.31±1.04	SRRI, 10 ml, qd	ST	4W	①②③⑦⑨⑩
Chen et al. (2016)	110	110	59/51	62/48	64.5±3.6	63.7±3.9	1∼6	1∼8	SRRI, 10 ml, qd	ST	4W	①②④⑤⑥⑦⑧⑩
Dong et al. (2023)	40	40	19/21	23/17	67.3±3.9	67.9±4.1	8.17±2.6	8.20±2.5	SRRI, 10 ml, qd	ST	2W	①②③④⑥⑨
Fan et al. (2017)	33	33	18/15	17/16	73.8±8.8	71.6±7.6	/	/	SRRI, 10 ml, qd	ST	2W	①②⑦
Guan. (2020)	50	50	31/19	23/27	64.14±2.13	64.25±2.11	12.71±0.54	12.78±0.92	SRRI, 10 ml, qd	ST	4W	①②③⑦⑧⑩
Hu et al. (2018)	50	50	29/21	33/17	63.57±5.24	63.22±5.52	4.21±2.51	4.23±2.56	SRRI, 10 ml, qd	ST	30d	①②③④⑤⑥⑦⑧⑩
Hua et al. (2017)	42	41	26/16	23/18	63.11±14.34	62.67±14.25	/	/	SRRI, 10 ml, qd	ST	4W	①②⑦⑨⑩
Jin and Li. (2016)	52	52	35/17	37/15	58.8±3.7	58.5±4.2	13.4±2.0	13.5±2.4	SRRI, 10 ml, qd	ST	10d	①②③⑦⑧
Lai et al. (2019)	35	35	19/16	20/15	55.37±5.09	56.52±4.86	4.87±2.23	4.63±2.08	SRRI, 10 ml, qd	ST	4W	①②⑦⑩
Li and Liang. (2020)	35	35	21/14	20/15	63.13±8.92	62.69±9.01	4.96±1.32	5.02±1.21	SRRI, 10 ml, qd	ST	30d	①②④⑤⑥⑦⑨
Lu et al. (2020)	25	25	14/11	15/10	60.59±6.48	60.38±6.57	4.26±1.36	4.32±1.42	SRRI, 10 ml, qd	ST	30d	①②③⑥⑦
Ning et al. (2017)	74	74	42/32	44/30	60.5±7.2	59.2±7.7	5.4±2.3	5.0±2.8	SRRI, 10 ml, qd	ST	2W	①②③④⑤⑦⑨⑩
Qi et al. (2016)	50	50	24/26	27/23	71.06±6.58	71.54±6.19	12.16±1.02	12.56±1.12	SRRI, 10 ml, qd	ST	4W	①②③⑦⑨
Shen et al. (2017)	50	50	34/16	32/18	70.23±3.45	70.43 ±3.65	2.65±1.68	2.35±1.75	SRRI, 10 ml, qd	ST	2W	①②③④⑤⑥⑨
Song et al. (2020)	40	40	24/16	26/14	65.89±7.45	66.04±7.22	/	/	SRRI, 10 ml, qd	ST	2W	①②③⑦⑩
Tian et al. (2019)	52	52	25/27	26/26	70.29±1.26	75.33±1.16	6.80±2.15	7.80±1.55	SRRI, 10 ml, qd	ST	20d	①②③⑦⑨⑩
Tian et al. (2017)	30	30	18/12	16/14	64.3±6.8	67.2±5.4	/	/	SRRI, 10 ml, qd	ST	10d	①⑦⑧⑩
Wang and Liu. (2018)	49	49	29/20	28/21	66.3±8.2	67.2±8.6	5.3±1.2	5.2±1.3	SRRI, 10 ml, qd	ST	2W	①②③⑤⑥⑦⑨⑩
Xie and Wen. (2016)	40	40	23/17	22/18	63.41±6.50	63.35±6.73	8.49±1.36	8.53±1.40	SRRI, 10 ml, qd	ST	1W	①⑦⑧
Xu. (2019)	40	40	24/16	25/15	58.25±6.50	57.47±6.30	4.74±0.51	4.62±0.43	SRRI, 10 ml, qd	ST	4W	①②⑦⑧
Yang. (2019)	23	23	12/11	13/10	63.5±6.8	63.0±6.6	/	/	SRRI, 10 ml, qd	ST	4W	①②③⑤⑥⑨
Zhang and Zhang. (2011)	29	29	16/13	14/15	70.04±5.94	71.43±7.91	4.47 ±1.86	4.86±1.17	SRRI, 10 ml, qd	ST	2W	①⑦
Zhang et al. (2018)	49	49	27/22	29/20	70.9±7.2	71.5±6.9	/	/	SRRI, 10 ml, qd	ST	10d	①②③⑥⑧
Zhang et al. (2020)	52	52	30/22	27/25	55.34±8.41	56.97±9.05	8.15±2.07	8.84±2.11	SRRI, 10 ml, qd	ST	20d	①②③⑦⑨
Zong et al. (2014)	74	72	39/35	37/35	67.37±9.83	65.78±10.35	8.25±3.30	7.75±3.50	SRRI, 10 ml, qd	ST	2W	①⑦⑧⑩

Note: C, control group; T, treatment group; M, male; F, female; d, days; W, Weeks; qd, quaque in die; ST, standard therapy per clinical guidelines; Outcomes: ①LVEF; ②LVEDD; ③LVESD; ④TNF-α; ⑤IL-6; ⑥hs-CRP; ⑦Clinical effectiveness; ⑧BNP; ⑨NT-pro BNP; ⑩Adverse reactions.

### 3.2 Risk of bias assessment

**FIGURE 2:**
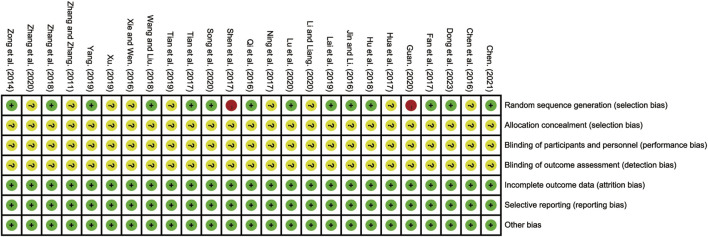
Bias risk assessment of included studies.

All 25 included RCTs adequately reported the randomization process. Fourteen studies employed the random number table method and were classified as low-risk RCTs (Chen, 2021; Dong et al., 2023; Fan et al., 2017; Hu et al., 2018; Jin and Li, 2016; Lai et al., 2019; Lu et al., 2020; Qi et al., 2016; Song et al., 2020; Tian et al., 2017; Wang and Liu, 2018; Yang, 2019; Zhang et al., 2018; Zong et al., 2014). One study used an odd-even grouping method (Shen et al., 2017), and another grouped participants based on treatment method (Guan, 2020); both were classified as high risk. The remaining nine studies did not provide sufficient details on their randomization methods and were categorized as having an unclear risk of bias (Chen et al., 2016; Hua et al., 2017; Li and Liang, 2020; Ning et al., 2017; Tian et al., 2019; Xie and Wen, 2016; Xu, 2019; Zhang and Zhang, 2011; Zhang et al., 2020). None of the included studies reported blinding or allocation concealment, resulting in these domains being designated as unclear risk. All studies reported complete outcome data and were considered low risk in this domain. No study explicitly reported other potential sources of bias, and thus these were classified as unclear risk. The detailed risk of bias assessment is illustrated in Figure 2 and Supplementary Material S3.

### 3.3 Primary outcomes

#### 3.3.1 LVEF

**FIGURE 3:**
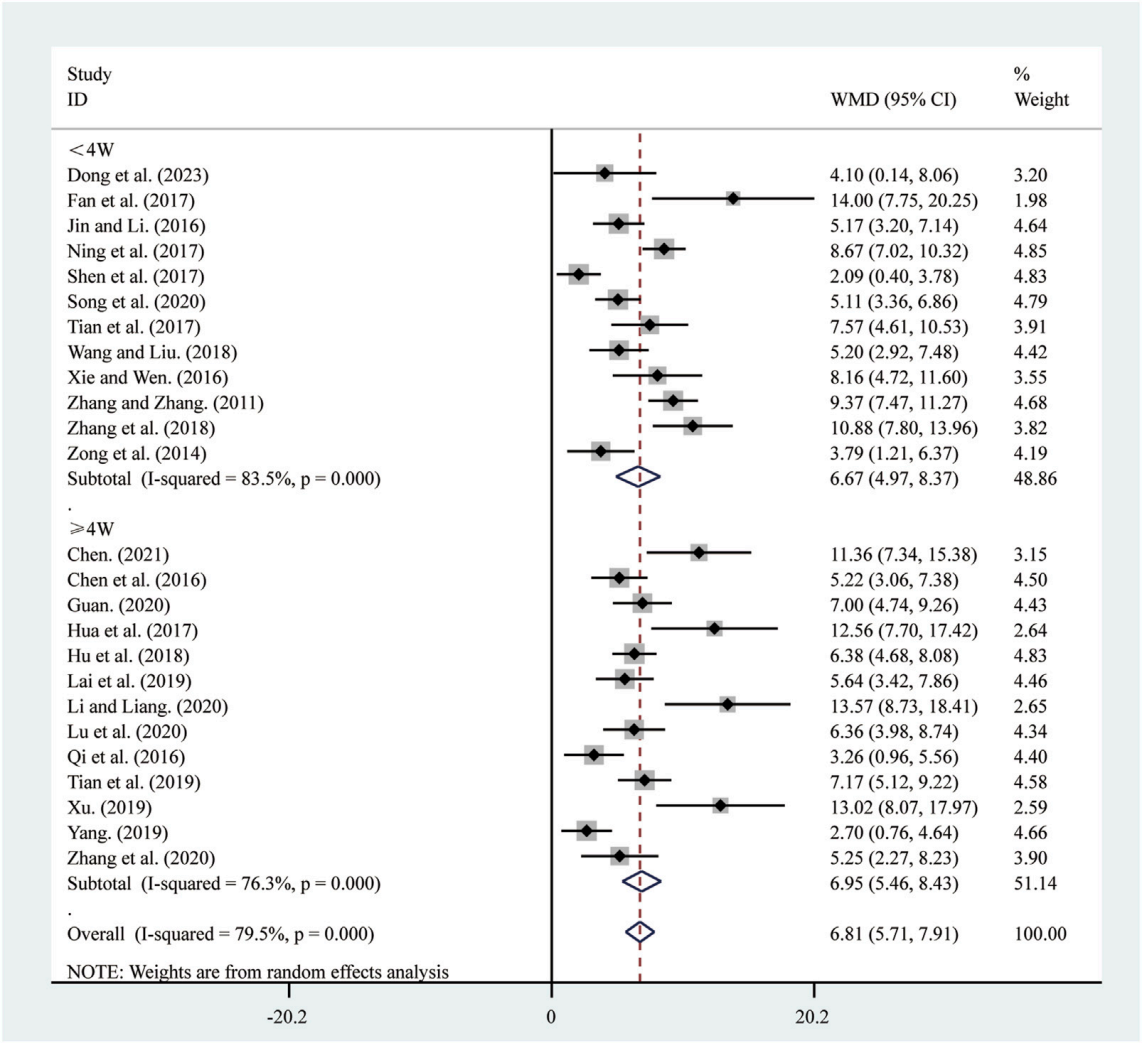
Forest plot for LVEF.

All 25 studies (Chen, 2021; Chen et al., 2016; Dong et al., 2023; Fan et al., 2017; Guan, 2020; Hu et al., 2018; Hua et al., 2017; Jin and Li, 2016; Lai et al., 2019; Li and Liang, 2020; Lu et al., 2020; Ning et al., 2017; Qi et al., 2016; Shen et al., 2017; Song et al., 2020; Tian et al., 2019; Tian et al., 2017; Wang and Liu, 2018; Xie and Wen, 2016; Xu, 2019; Yang, 2019; Zhang and Zhang, 2011; Zhang et al., 2018; Zhang et al., 2020; Zong et al., 2014) reported on LVEF. A random-effects model was used to pool the effect sizes due to significant heterogeneity (*I* ^*2*^ = 79.5%, *P* = 0.000). Compared to ST, SRRI significantly improved LVEF (MD = 6.81, 95% CI: 5.71 to 7.91, *P* = 0.000, Figure 3). Subgroup analysis based on the duration of SRRI treatment revealed significant differences between SRRI and ST: less than 4 weeks (MD = 6.67, 95% CI: 4.97 to 8.37, *P* = 0.000, Figure 3) and more than 4 weeks (MD = 6.95, 95% CI: 5.46 to 8.43, *P* = 0.000, Figure 3).

#### 3.3.2 LVEDD

**FIGURE 4:**
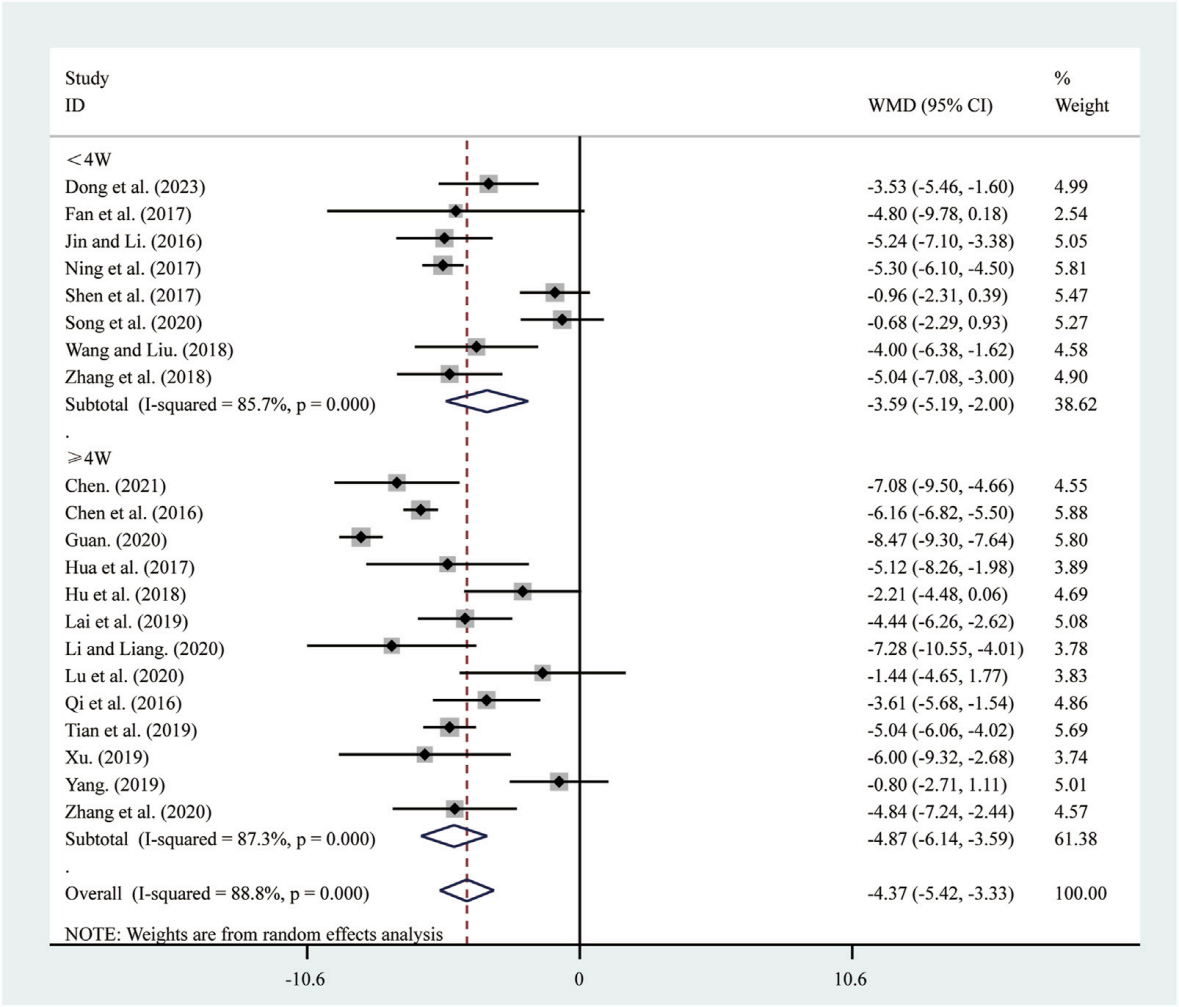
Forest plot for LVEDD.

21 studies (Chen, 2021; Chen et al., 2016; Dong et al., 2023; Fan et al., 2017; Guan, 2020; Hu et al., 2018; Hua et al., 2017; Jin and Li, 2016; Lai et al., 2019; Li and Liang, 2020; Lu et al., 2020; Ning et al., 2017; Qi et al., 2016; Shen et al., 2017; Song et al., 2020; Tian et al., 2019; Wang and Liu, 2018; Xu, 2019; Yang, 2019; Zhang et al., 2018; Zhang et al., 2020) reported on LVEDD. A random-effects model was used to pool the effect sizes due to significant heterogeneity (*I* ^2^ = 88.8%, *P* = 0.000). Compared to ST, SRRI significantly reduced LVEDD (MD = −4.37, 95% CI: −5.42 to −3.33, *P* = 0.000, Figure 4). Subgroup analysis based on the duration of SRRI treatment revealed significant differences between SRRI and ST: less than 4 weeks (MD = −3.59, 95% CI: −5.19 to −2.00, *P* = 0.000, Figure 4) and more than 4 weeks (MD = −4.87, 95% CI: −6.14 to −3.59, *P* = 0.000, Figure 4).

#### 3.3.3 LVESD

**FIGURE 5:**
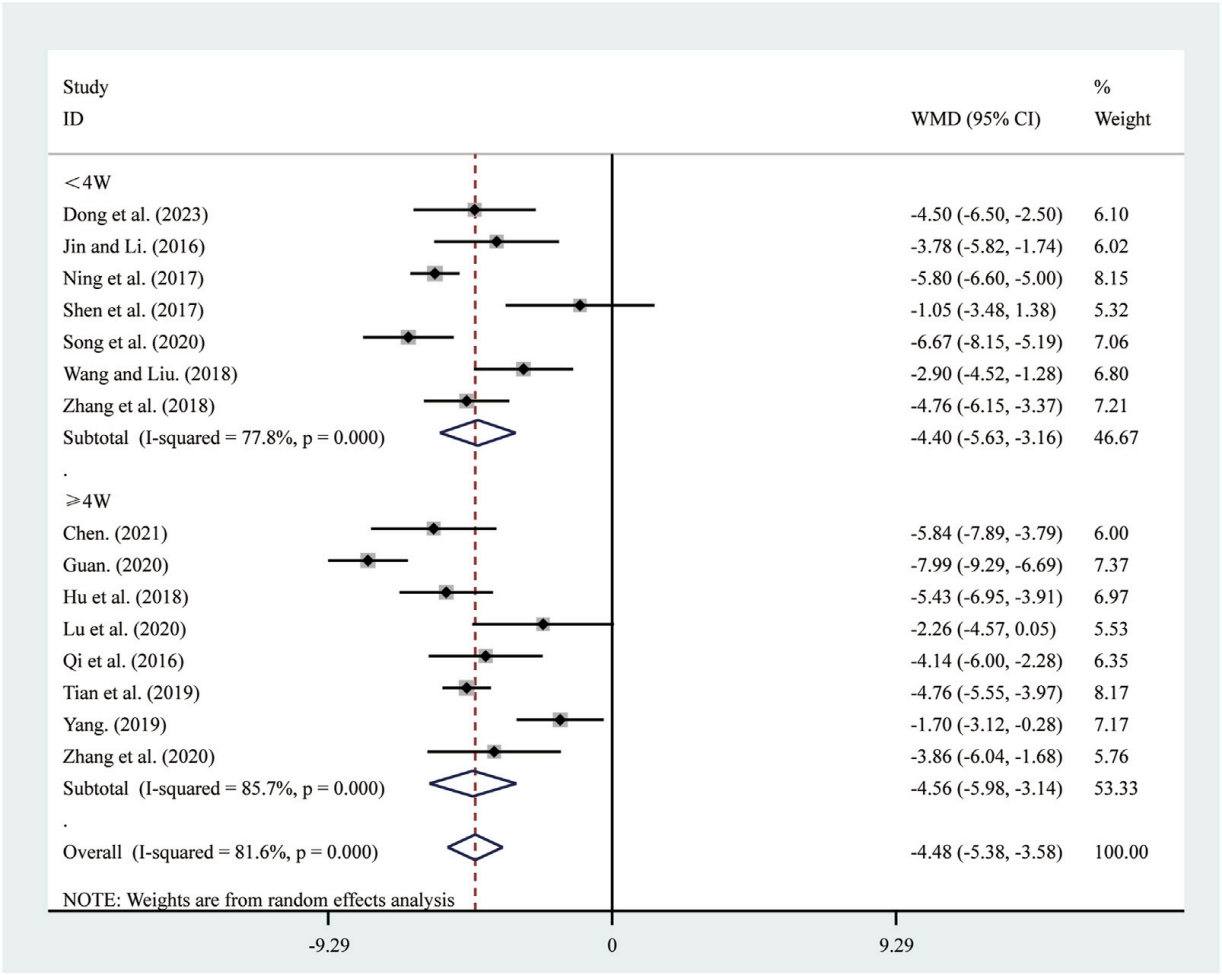
Forest plot for LVESD.

15 studies (Chen, 2021; Dong et al., 2023; Guan, 2020; Hu et al., 2018; Jin and Li, 2016; Lu et al., 2020; Ning et al., 2017; Qi et al., 2016; Shen et al., 2017; Song et al., 2020; Tian et al., 2019; Wang and Liu, 2018; Yang, 2019; Zhang et al., 2018; Zhang et al., 2020) reported on LVESD. A random-effects model was used to pool the effect sizes due to significant heterogeneity (*I* ^2^ = 81.6%, *P* = 0.000). Compared to ST, SRRI significantly reduced LVESD (MD = −4.48, 95% CI: −5.38 to −3.58, *P* = 0.000, Figure 5). Subgroup analysis based on the duration of SRRI treatment revealed significant differences between SRRI and ST: less than 4 weeks (MD = −4.40, 95% CI: −5.63 to −3.16, *P* = 0.000, Figure 5) and more than 4 weeks (MD = −4.56, 95% CI: −5.98 to −3.14, *P* = 0.000, Figure 5).

#### 3.3.4 TNF-α

**FIGURE 6:**
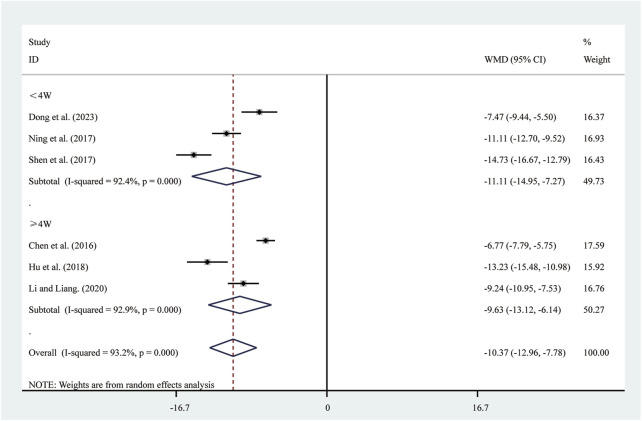
Forest plot for TNF-α.

Six studies (Chen et al., 2016; Dong et al., 2023; Hu et al., 2018; Li and Liang, 2020; Ning et al., 2017; Shen et al., 2017) reported on TNF-α. A random-effects model was used to pool the effect sizes due to significant heterogeneity (*I* ^2^ = 93.2%, *P* = 0.000). Compared to ST, SRRI significantly reduced TNF-α (MD = −10.37, 95% CI: −12.96 to −7.78, *P* = 0.000, Figure 6). Subgroup analysis based on the duration of SRRI treatment revealed significant differences between SRRI and ST: less than 4 weeks (MD = −11.11, 95% CI: −14.95 to −7.27, *P* = 0.000, Figure 6) and more than 4 weeks (MD = −9.63, 95% CI: −13.12 to −6.14, *P* = 0.000, Figure 6).

#### 3.3.5 IL-6

**FIGURE 7:**
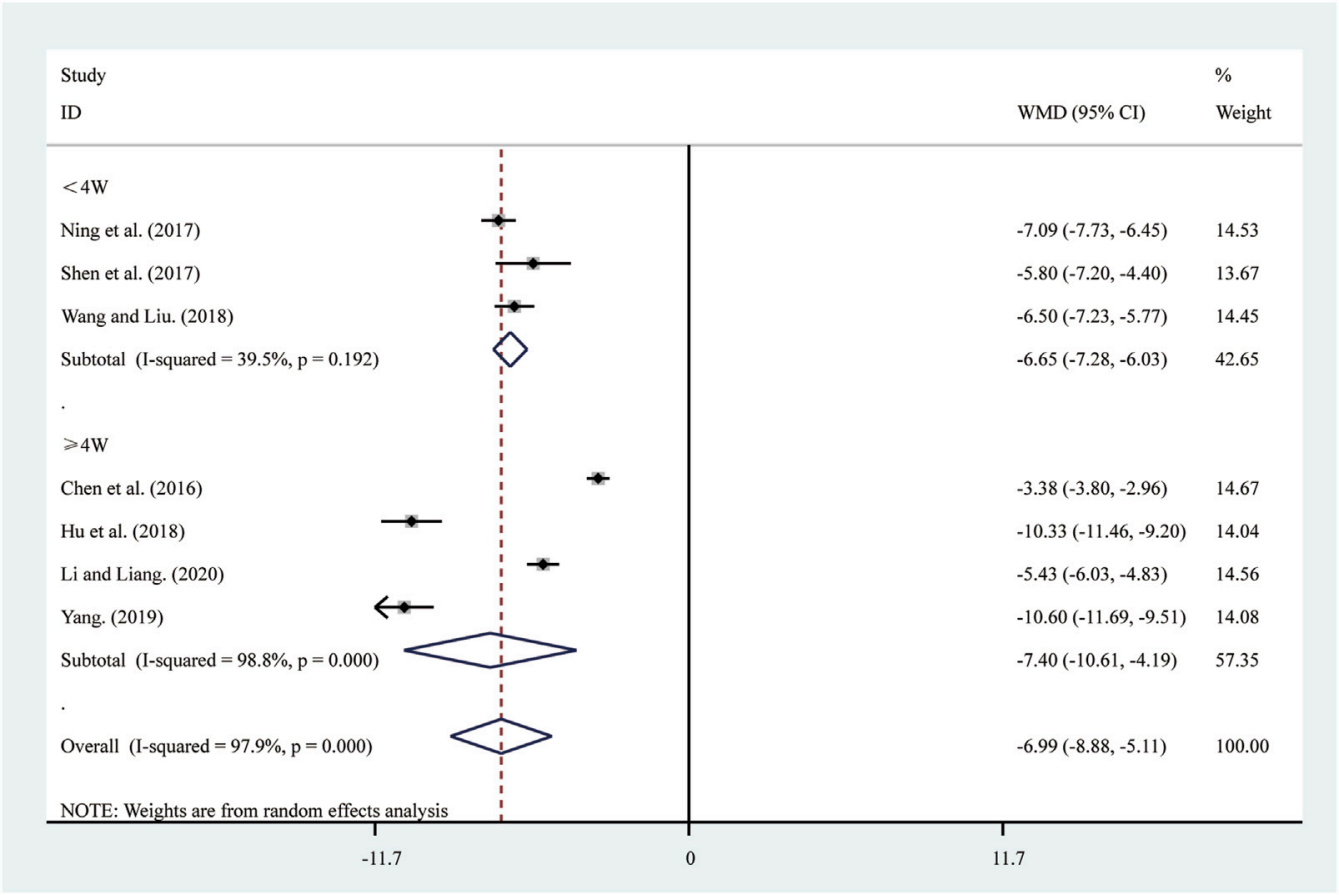
Forest plot for IL-6.

Seven studies (Chen et al., 2016; Hu et al., 2018; Li and Liang, 2020; Ning et al., 2017; Shen et al., 2017; Wang and Liu, 2018; Yang, 2019) reported on IL-6. A random-effects model was used to pool the effect sizes due to significant heterogeneity (*I* ^2^ = 97.9%, *P* = 0.000). Compared to ST, SRRI significantly reduced IL-6 (MD = −6.99, 95% CI: −8.88 to −5.11, *P* = 0.000, Figure 7). Subgroup analysis based on the duration of SRRI treatment revealed significant differences between SRRI and ST: less than 4 weeks (MD = −6.65, 95% CI: −7.28 to −6.03, *P* = 0.000, Figure 7) and more than 4 weeks (MD = −7.40, 95% CI: −10.61 to −4.19, *P* = 0.000, Figure 7).

#### 3.3.6 hs-CRP

**FIGURE 8:**
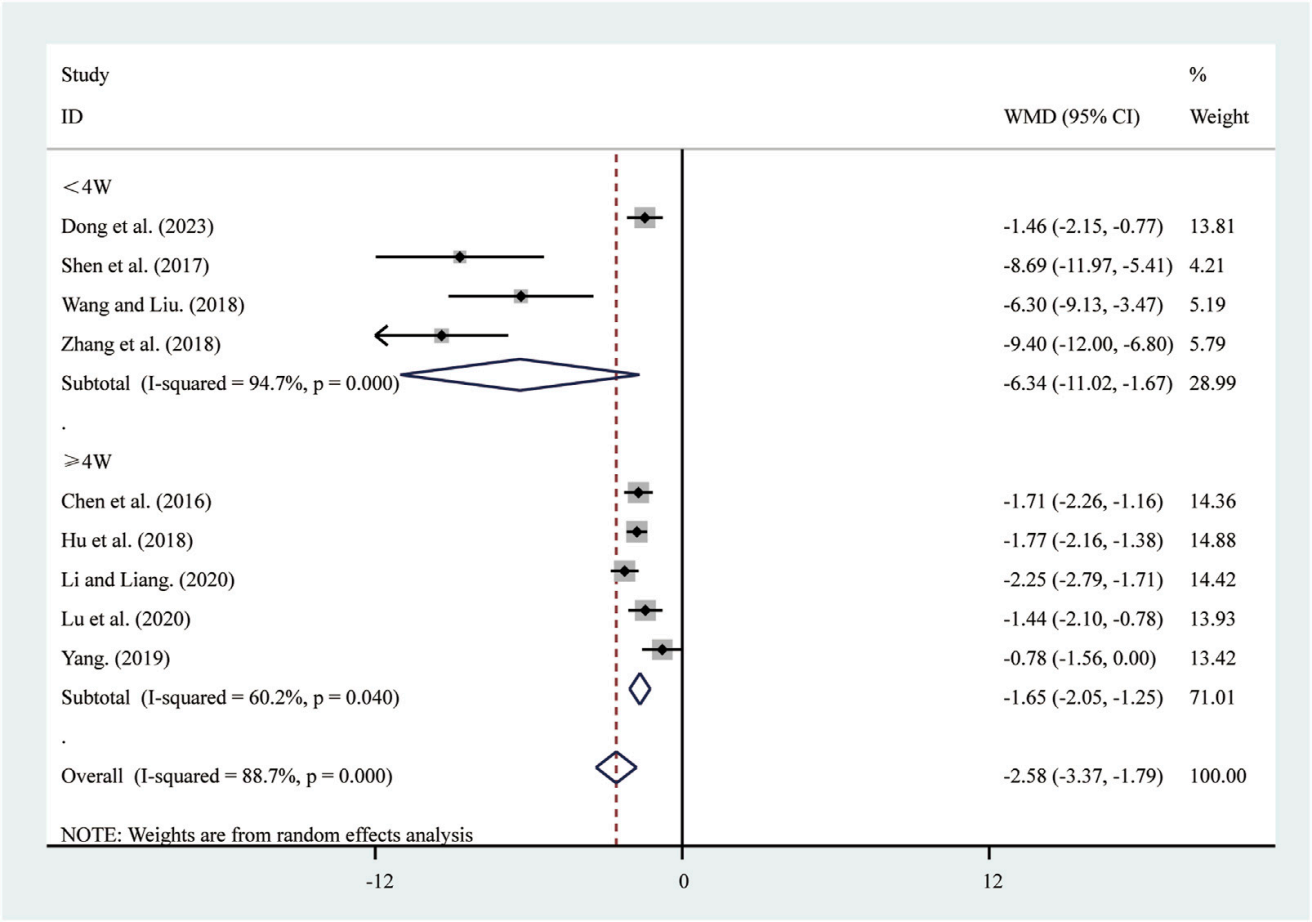
Forest plot for hs-CRP.

Nine studies (Chen et al., 2016; Dong et al., 2023; Hu et al., 2018; Li and Liang, 2020; Lu et al., 2020; Shen et al., 2017; Wang and Liu, 2018; Yang, 2019; Zhang et al., 2018) reported on hs-CRP. A random-effects model was used to pool the effect sizes due to significant heterogeneity (*I* ^2^ = 88.7%, *P* = 0.000). Compared to ST, SRRI significantly reduced hs-CRP (MD = −2.58, 95% CI: −3.37 to −1.79, *P* = 0.000, Figure 8). Subgroup analysis based on the duration of SRRI treatment revealed significant differences between SRRI and ST: less than 4 weeks (MD = −6.34, 95% CI: −11.02 to −1.67, *P* = 0.000, Figure 8) and more than 4 weeks (MD = −1.65, 95% CI: −2.05 to −1.25, *P* = 0.000, Figure 8).

### 3.4 Secondary outcomes

#### 3.4.1 Clinical effectiveness

**FIGURE 9:**
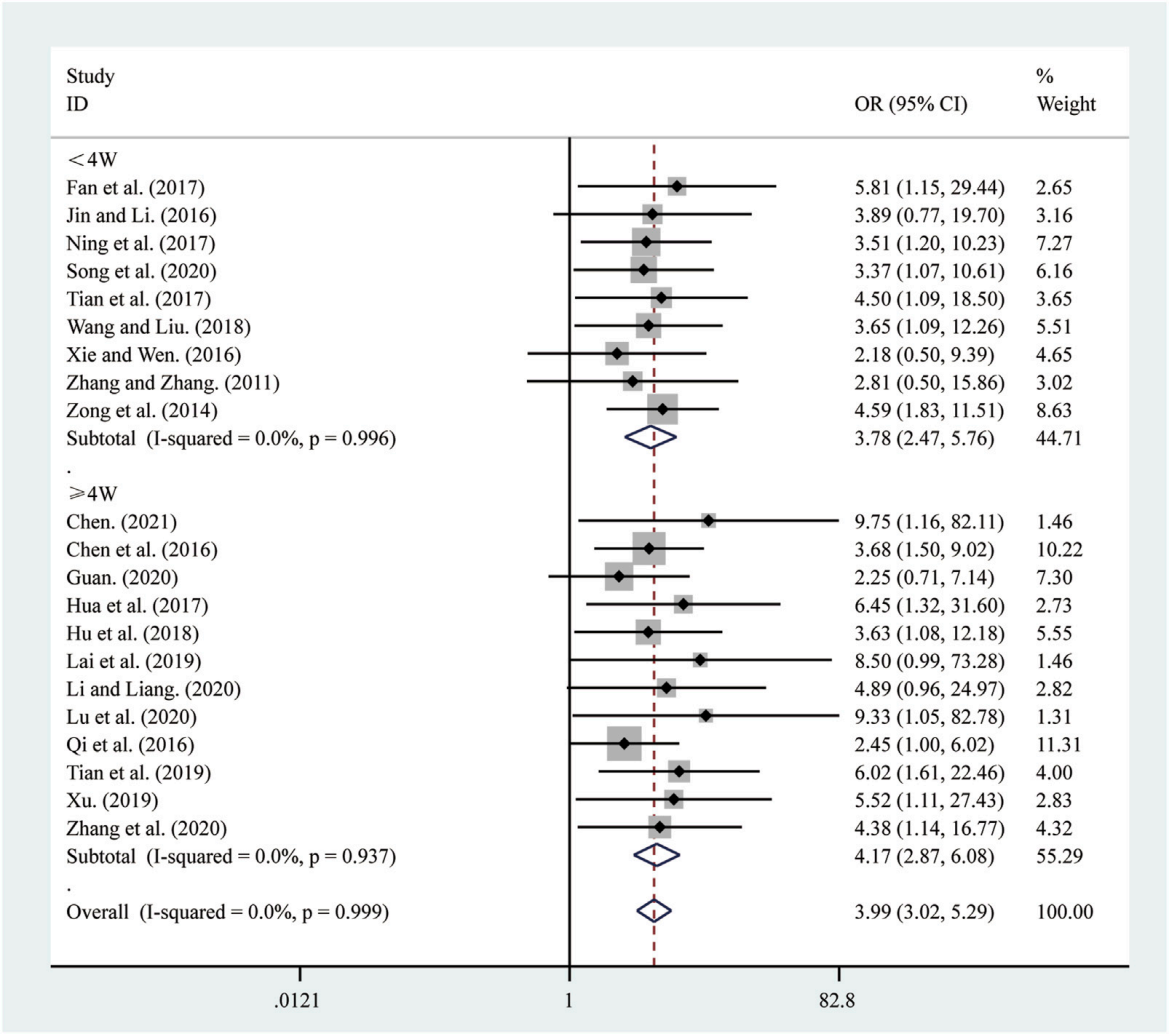
Forest plot for clinical effectiveness.

21 studies (Chen, 2021; Chen et al., 2016; Fan et al., 2017; Guan, 2020; Hu et al., 2018; Hua et al., 2017; Jin and Li, 2016; Lai et al., 2019; Li and Liang, 2020; Lu et al., 2020; Ning et al., 2017; Qi et al., 2016; Song et al., 2020; Tian et al., 2019; Tian et al., 2017; Wang and Liu, 2018; Xie and Wen, 2016; Xu, 2019; Zhang and Zhang, 2011; Zhang et al., 2020; Zong et al., 2014) reported on clinical effectiveness. A fixed-effects model was used to pool the effect sizes due to low heterogeneity (*I* ^*2*^ = 0.0%, *P =* 0.999). Compared to ST, SRRI significantly improved clinical effectiveness (RR = 3.99, 95% CI: 3.02 to 5.29, *P* = 0.000, Figure 9). A subgroup analysis based on the duration of SRRI treatment revealed significant differences between SRRI and ST: less than 4 weeks (RR = 3.78, 95% CI: 2.47 to 5.76, *P* = 0.000, Figure 9) and more than 4 weeks (RR = 4.17, 95% CI: 2.87 to 6.08, *P* = 0.000, Figure 9).

#### 3.4.2 BNP

**FIGURE 10:**
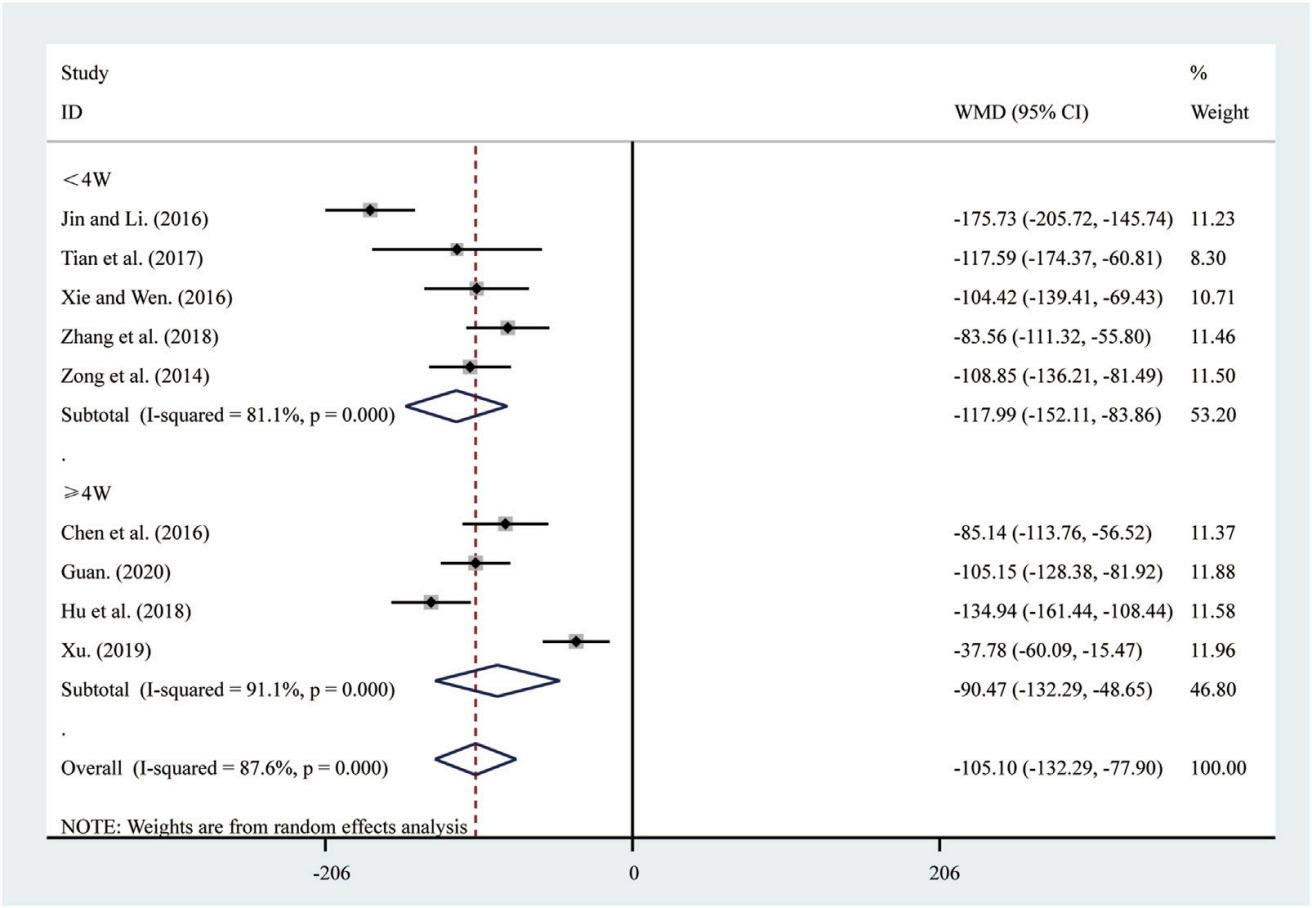
Forest plot for BNP.

Nine studies (Chen et al., 2016; Guan, 2020; Hu et al., 2018; Jin and Li, 2016; Tian et al., 2017; Xie and Wen, 2016; Xu, 2019; Zhang et al., 2018; Zong et al., 2014) reported on BNP. A random-effects model was used to pool the effect sizes due to significant heterogeneity (*I* ^2^ = 87.6%, *P* = 0.000). Compared to ST, SRRI significantly reduced BNP (MD = −105.10, 95% CI: −132.29 to −77.90, *P* = 0.000, Figure 10). A subgroup analysis based on the duration of SRRI treatment revealed significant differences between SRRI and ST: less than 4 weeks (MD = −117.99, 95% CI: −152.11 to −83.86, *P* = 0.000, Figure 10) and more than 4 weeks (MD = −90.47, 95% CI: −132.29 to −48.65, *P* = 0.000, Figure 10).

#### 3.4.3 NT-pro BNP

**FIGURE 11:**
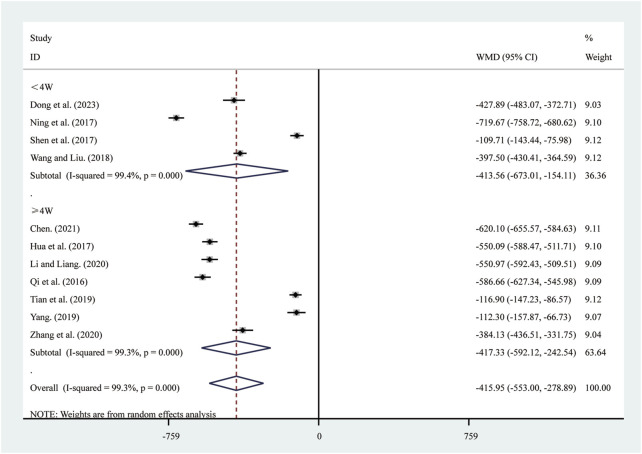
Forest plot for NT-pro BNP.

11 studies (Chen, 2021; Dong et al., 2023; Hua et al., 2017; Li and Liang, 2020; Ning et al., 2017; Qi et al., 2016; Shen et al., 2017; Tian et al., 2019; Wang and Liu, 2018; Yang, 2019; Zhang et al., 2020) reported on NT-pro BNP. A random-effects model was used to pool the effect sizes due to significant heterogeneity (*I* ^2^ = 99.3%, *P* = 0.000). Compared to ST, SRRI significantly reduced NT-pro BNP (MD = −415.95, 95% CI: −553.00 to −278.89, *P* = 0.000, Figure 11). A subgroup analysis based on the duration of SRRI treatment revealed significant differences between SRRI and ST: less than 4 weeks (MD = −413.56, 95% CI: −673.01 to −154.11, *P* = 0.000, Figure 11) and more than 4 weeks (MD = −417.33, 95% CI: −592.12 to −242.54, *P* = 0.000, Figure 11).

#### 3.4.4 Adverse reactions

**FIGURE 12:**
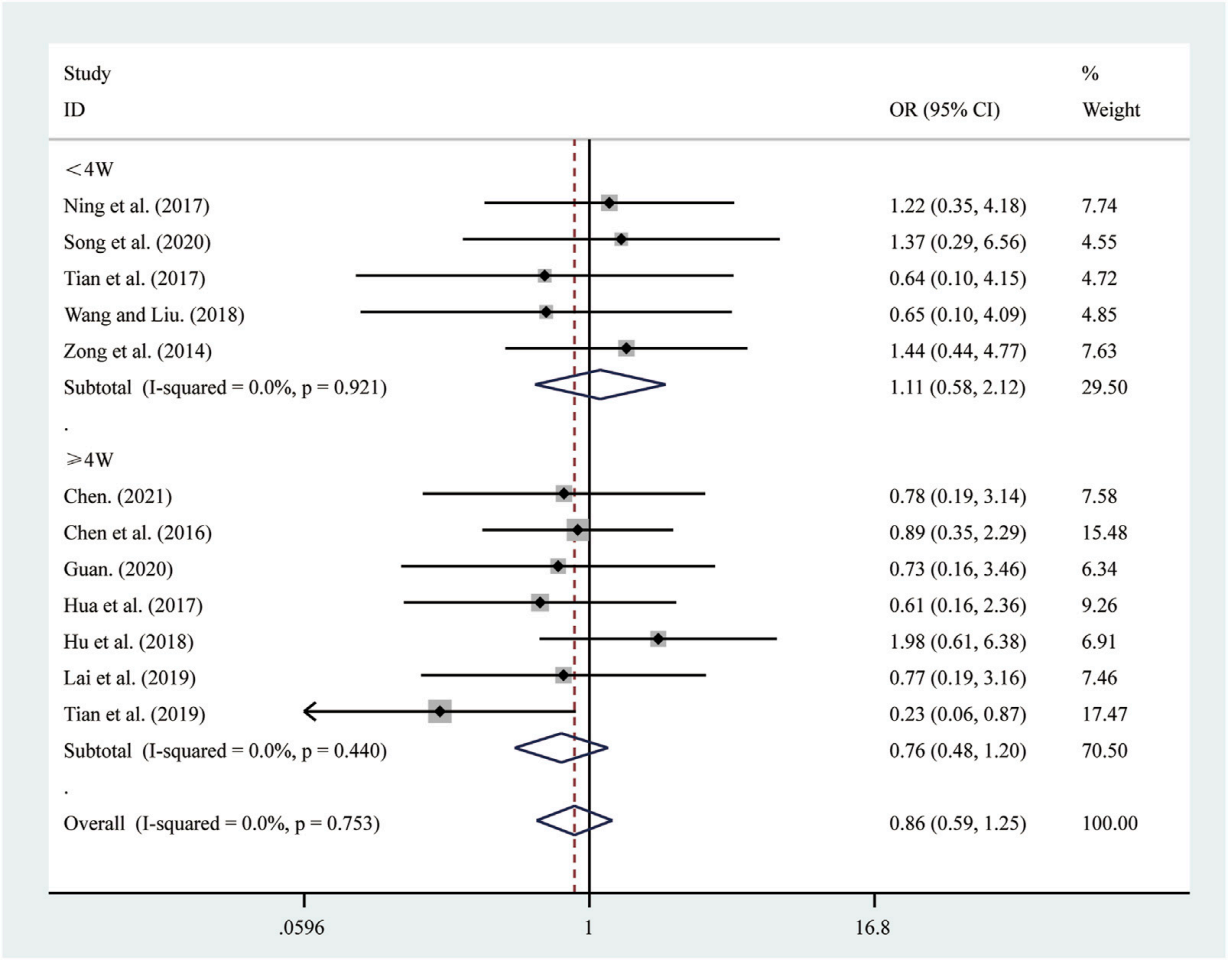
Forest plot for adverse reactions.

12 studies (Chen, 2021; Chen et al., 2016; Guan, 2020; Hu et al., 2018; Hua et al., 2017; Lai et al., 2019; Ning et al., 2017; Song et al., 2020; Tian et al., 2019; Tian et al., 2017; Wang and Liu, 2018; Zong et al., 2014) reported on adverse reactions. A fixed-effects model was used to pool the effect sizes due to low heterogeneity (*I* ^*2*^ = 0.0%, *P =* 0.753). Compared to ST, SRRI did not increase adverse reactions (RR = 0.86, 95% CI: 0.59 to 1.25, *P* = 0.440, Figure 12). A subgroup analysis based on the duration of SRRI treatment revealed no significant difference between SRRI and ST: less than 4 weeks (RR = 1.11, 95% CI: 0.58 to 2.12, *P* = 0.742, Figure 12) and more than 4 weeks (RR = 0.76, 95% CI: 0.48 to 1.20, *P* = 0.239, Figure 12). Detailed information on adverse reactions can be found in Supplementary Material S4.

### 3.5 Sensitivity analysis

**FIGURE 13:**
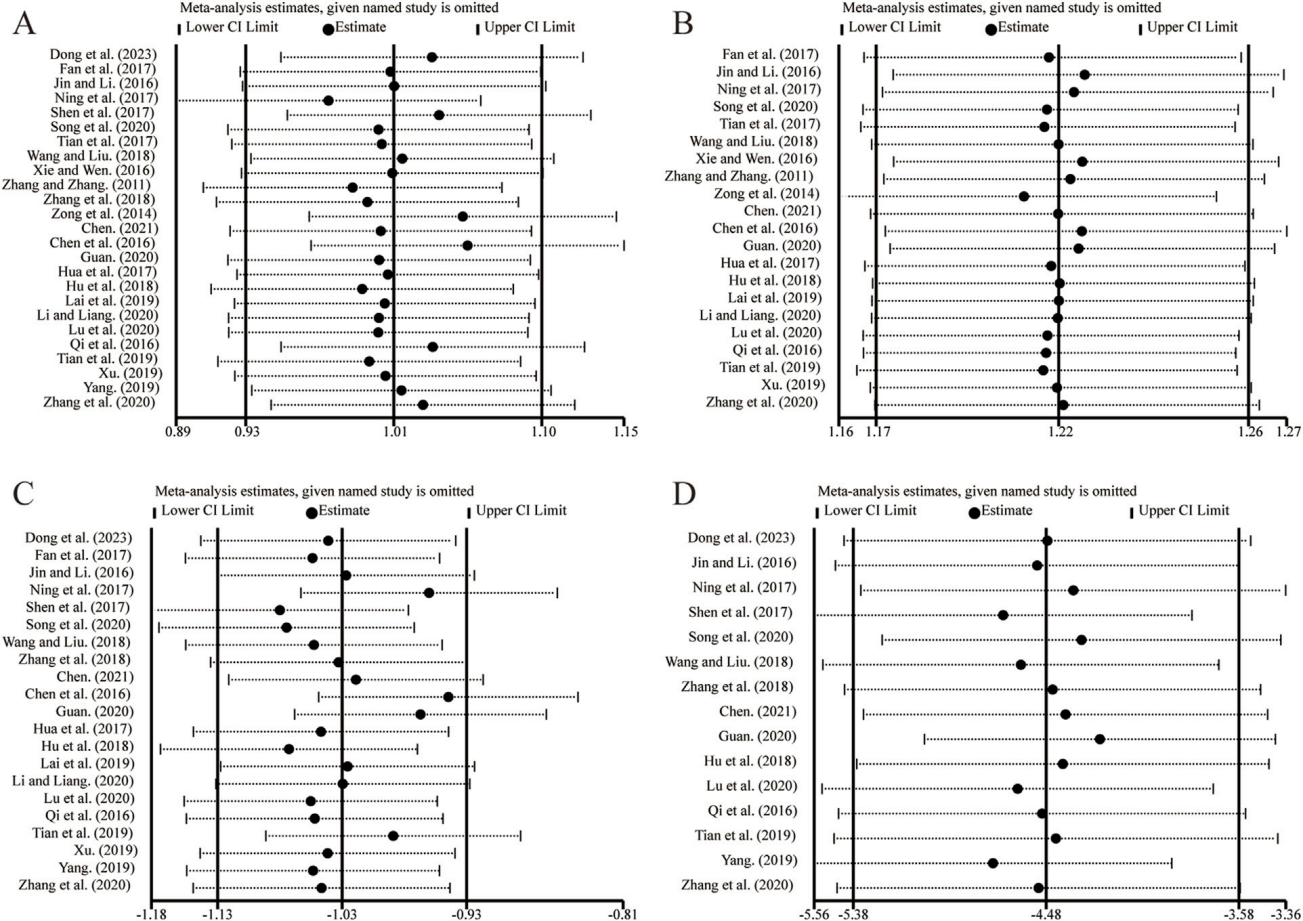
The results of sensitivity analysis. **(A)** LVEF. **(B)** Clinical effectiveness. **(C)** LVEDD. **(D)** LVESD.

Sensitivity analysis was conducted by sequentially excluding individual studies to evaluate their impact on the overall pooled results. Analyses were performed for outcome measures with more than ten included studies. The results demonstrated that removing any single study did not alter the combined results for LVEF (Figure 13A), clinical effectiveness (Figure 13B), LVEDD (Figure 13C), and LVESD (Figure 13D). These findings suggest that the pooled results are robust and reliable.

### 3.6 Publication bias

**FIGURE 14:**
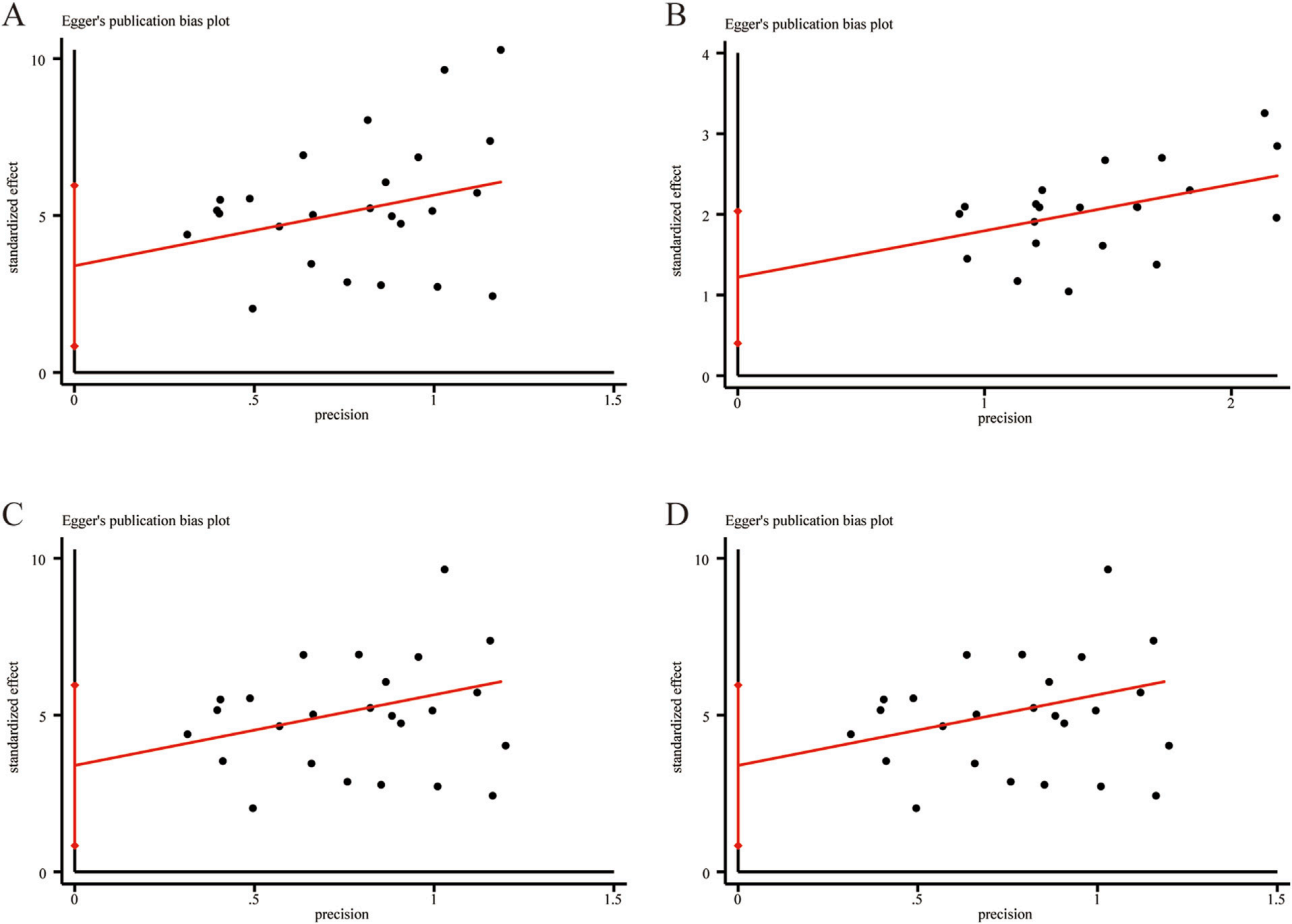
Egger’s publication funnel plot. **(A)** LVEF. **(B)** Clinical effectiveness. **(C)** LVEDD. **(D)** LVESD.

Egger’s test was employed to assess publication bias for outcome measures with more than ten included studies. The results indicated no significant publication bias for LVEF (Figure 14A *, P* = 0.112), clinical effectiveness (Figure 14B *, P* = 0.126), LVEDD (Figure 14C *, P* = 0.260), and LVESD (Figure 14D *, P* = 0.141).

## 4 Discussion

### 4.1 Mechanisms of SRRI in cardioprotection

The cardioprotective effects of SRRI may be attributed to its bioactive compound, salidroside (*R. rosea* L.), which modulates multiple molecular pathways involved in HF pathophysiology. These mechanisms include the regulation of inflammatory responses, inhibition of oxidative stress, stimulation of mitochondrial biogenesis, modulation of autophagy, and prevention of cardiomyocyte apoptosis (Zong et al., 2023). Salidroside inhibits ischemia-reperfusion-induced cardiomyocyte apoptosis by suppressing the JNK signaling pathway (Sun et al., 2012). It counteracts hypoxia-induced cardiomyocyte necrosis and apoptosis by upregulating the expression of HIF-1α protein and inducing its translocation, which in turn upregulates the expression levels of the downstream target VEGF (Zhang et al., 2009). Pre-treatment with salidroside significantly upregulates the Bcl-2/Bax ratio, increases AKT phosphorylation, and reduces the activation of Caspase-3, thus inhibiting apoptosis and maintaining mitochondrial membrane potential (Zhong et al., 2010). Furthermore, salidroside enhances autophagy induced by oxidized low-density lipoprotein in endothelial cells by increasing the expression levels of SIRT1 and FoxO1, thereby reducing oxidative stress in endothelial cells and exerting its endothelial protective effect (Zhu et al., 2019). Salidroside can also induce autophagy to protect vascular endothelial cells from oxidative stress-induced apoptosis, primarily through the AMPK-mTOR pathway, increasing AMPK phosphorylation while decreasing mTOR phosphorylation (Zheng et al., 2017). Additionally, salidroside regulates K+ and Ca2+ channels and activates the PI3K/AKT signaling pathway to inhibit atrial fibrillation and atrial fibrosis, thus suppressing atrial arrhythmias in heart failure models (Liu et al., 2016).

### 4.2 Summary of findings

This meta-analysis is the first to comprehensively review the effects of SRRI on LVR and inflammatory mediators in patients with HFrEF. A total of 25 RCTs involving 2,325 patients were included. The meta-analysis findings demonstrated the following: (1) SRRI significantly improved LVR in HFrEF patients, evidenced by increased LVEF and decreased LVEDD and LVESD; (2) SRRI notably reduced inflammation, as indicated by lowered levels of inflammatory mediators such as TNF-α, IL-6, and hs-CRP; (3) SRRI exhibited higher clinical effectiveness and a good safety profile. These results strongly suggest that SRRI can effectively enhance LVR, reduce inflammatory mediators, and improve overall clinical effectiveness in HFrEF patients.

Sensitivity analyses confirmed the robustness and reliability of the meta-analysis results. No significant publication bias was detected using Egger’s test. Subgroup analyses, conducted based on the hypothesis that treatment duration may influence therapeutic outcomes, revealed distinct patterns of improvement. Patients receiving SRRI for ≤4 weeks exhibited more pronounced reductions in inflammatory markers (TNF-α, hs-CRP) and BNP, suggesting that the early-phase effects of SRRI may be primarily associated with its anti-inflammatory and circulatory-enhancing properties. In contrast, patients treated for >4 weeks demonstrated greater improvements in ventricular remodeling indicators (LVEF, LVEDD, LVESD), IL-6, clinical effectiveness, and NT-pro BNP, indicating that prolonged treatment may be necessary for structural cardiac improvements and sustained myocardial functional recovery.

In traditional Chinese medicine (TCM) research, a 4-week treatment course is often employed to determine short-term clinical effectiveness and guide subsequent adjustments in therapy. This distinction between short-term and long-term effects is consistent with the progressive nature of ventricular remodeling, which generally requires extended therapeutic intervention to observe significant structural modifications. The greater improvements in LVEF and LV dimensions beyond 4 weeks may be attributed to cumulative effects on mitochondrial protection, myocardial autophagy regulation, and inhibition of apoptotic pathways, which require longer durations to manifest. Although the differences between the two subgroups were not substantial, this analysis helped identify potential time-dependent therapeutic effects of SRRI and contributed to a more refined interpretation of the pooled results. These findings provide valuable insights into the optimal duration of SRRI treatment in HFrEF patients and highlight the need for longer-term studies to further validate its sustained clinical benefits and safety profile.

### 4.3 Comparison with previous studies

Previous meta-analyses by Zhang et al. (2023), Ou et al. (2019) investigated the clinical effectiveness of SRRI in treating HFrEF patients, but neither focused on its impact on LVR or inflammatory mediators. Zhang et al. primarily assessed the overall clinical effectiveness and evidence quality of combining SRRI with Western medicine for heart failure treatment. Ou et al. centered on short-term clinical outcomes, including clinical effectiveness and adverse events, associated with SRRI used in combination with Western medicine. Their findings demonstrated that SRRI, when combined with standard therapy, significantly improved clinical effectiveness in HFrEF patients. However, neither study delved into the mechanisms underlying these outcomes, particularly concerning LVR and inflammatory mediators.

In contrast, our study is the first to comprehensively assess SRRI’s effects on LVR indicators (LVEF, LVEDD, and LVESD) and its ability to suppress inflammatory mediators such as TNF-α, IL-6, and hs-CRP. While conventional HFrEF treatments (e.g., ACE inhibitors/ARBs, β-blockers, diuretics) primarily aim to control clinical symptoms and manage ventricular remodeling, SRRI offers additional advantages by addressing both LVR improvement and inflammation reduction. Moreover, conventional treatments often come with side effects such as cough, conduction abnormalities, electrolyte imbalances, and gastrointestinal discomfort (Kittleson, 2024). Our meta-analysis underscores the significant effects of SRRI in improving LVR and reducing inflammatory responses, accompanied by a favorable safety profile. Prolonged use of SRRI could potentially provide even greater clinical benefits. Regarding safety, SRRI demonstrated a strong safety profile across the included studies, reinforcing its potential as a safer alternative or adjunct therapy for HFrEF patients. However, detailed data on adverse events remain limited, highlighting the need for future research to focus on the long-term safety of SRRI.

### 4.4 Strengths and limitations

LVR is a critical pathological basis of HFrEF, with inflammation playing a significant role in exacerbating this process (Ministrini and Camici, 2024; Yang et al., 2022). When exposed to exogenous or endogenous stimuli, an inflammatory response is triggered, leading to the release of pro-inflammatory cytokines. These cytokines can activate matrix degradation processes and increase matrix metalloproteinase activity, initiating extracellular matrix (ECM) degradation (Rodrigues et al., 2024). Concurrently, inflammatory cytokines stimulate fibroblast proliferation, causing excessive ECM deposition in the cardiac stroma (Rodrigues et al., 2024). Myocardial fibrosis induced by inflammation results in myocardial stiffness and apoptosis, impairing both systolic and diastolic functions, thereby triggering ventricular remodeling and eventually heart failure (Wu et al., 2021). Key inflammatory markers such as TNF-α, IL-6, and hs-CRP are closely linked to HF. Effectively suppressing the inflammatory response is crucial for improving ventricular remodeling and slowing the progression of HFrEF (Cho et al., 2020). This meta-analysis is the first to specifically explore the effects of SRRI on LVR and inflammatory mediators in HFrEF patients. In addition, we included subgroup analyses based on treatment duration, which has not been extensively examined in prior studies, providing valuable insights into the potential time-dependent therapeutic effects of SRRI.

Nonetheless, this study has limitations. First, all included studies were conducted in China, which limits the generalizability of the findings to other populations. While the results suggest potential benefits of SRRI for HFrEF, the applicability of these findings to diverse ethnic and geographic populations remains uncertain. Second, the risk of bias analysis revealed several methodological shortcomings, particularly concerning allocation concealment and blinding. Many included studies did not adequately report their randomization methods or whether blinding was implemented, raising concerns about potential selection and performance biases. The absence of these methodological safeguards may have affected the validity and reliability of the findings. Third, the long-term safety profile of SRRI remains unclear due to insufficient reporting of adverse events in the included studies. Although no significant increase in adverse reactions was observed in the short term, the lack of detailed long-term safety data highlights the need for further studies with extended follow-up periods to assess its safety profile. Fourth, the lack of follow-up data in the included studies restricted the ability to evaluate the sustained effects of SRRI. Since HFrEF is a chronic condition requiring prolonged treatment, it remains uncertain whether the observed benefits persist over extended periods. Fifth, while this meta-analysis included inflammatory biomarkers such as TNF-α, IL-6, and hs-CRP, the limited number of studies reporting these outcomes reduces the strength of the evidence supporting the anti-inflammatory effects of SRRI. More high-quality RCTs are needed to confirm its role in modulating inflammation and its mechanistic impact on HFrEF.

### 4.5 Implication

To build on the findings of this meta-analysis, several implications for future research are evident. First, conducting multicenter clinical trials involving diverse populations is necessary to confirm and generalize the effectiveness of SRRI in treating HFrEF beyond the currently studied Chinese population. Second, rigorous methodological standards, particularly in terms of randomization, allocation concealment, and blinding, should be strictly followed to minimize bias and enhance the reliability of findings. Third, comprehensive safety evaluations, including detailed adverse event monitoring and pharmacokinetic studies, are essential to establish the long-term risk-benefit profile of SRRI in HFrEF management. Fourth, extended follow-up periods are necessary to assess the sustained effects of SRRI. Understanding whether its beneficial effects on left ventricular remodeling and inflammatory mediators persist over time will provide insight into its long-term therapeutic value, particularly in reducing rehospitalization rates and improving quality of life. Fifth, while this meta-analysis suggests that SRRI has anti-inflammatory properties, the limited number of studies reporting inflammatory biomarkers weakens the strength of evidence supporting this mechanism. High-quality RCTs should further investigate its impact on key inflammatory pathways, such as TNF-α, IL-6, and hs-CRP, to clarify its role in HFrEF management.

## 5 Conclusion

Current evidence supports that SRRI can effectively improve LVR, enhance cardiac function, and reduce inflammatory mediators in HFrEF patients, while maintaining a favorable safety profile. However, due to the low evidence levels and significant heterogeneity, particularly in the assessment of inflammatory mediators, future research should focus on high-quality RCTs to further substantiate these conclusions.

## Data Availability

The original contributions presented in the study are included in the article/Supplementary Material, further inquiries can be directed to the corresponding authors.
